# Clinical Decision Support and Natural Language Processing in Medicine: Systematic Literature Review

**DOI:** 10.2196/55315

**Published:** 2024-09-30

**Authors:** Hans Eguia, Carlos Luis Sánchez-Bocanegra, Franco Vinciarelli, Fernando Alvarez-Lopez, Francesc Saigí-Rubió

**Affiliations:** 1 SEMERGEN New Technologies Working Group Madrid Spain; 2 Faculty of Health Sciences Universitat Oberta de Catalunya (UOC) Barcelona Spain; 3 Emergency Hospital Clemente Álvarez Rosario (Santa Fe) Argentina; 4 Faculty of Health Sciences Universidad de Manizales Manizales Colombia

**Keywords:** artificial intelligence, AI, natural language processing, clinical decision support system, CDSS, health recommender system, clinical information extraction, electronic health record, systematic literature review, patient, treatment, diagnosis, health workers

## Abstract

**Background:**

Ensuring access to accurate and verified information is essential for effective patient treatment and diagnosis. Although health workers rely on the internet for clinical data, there is a need for a more streamlined approach.

**Objective:**

This systematic review aims to assess the current state of artificial intelligence (AI) and natural language processing (NLP) techniques in health care to identify their potential use in electronic health records and automated information searches.

**Methods:**

A search was conducted in the PubMed, Embase, ScienceDirect, Scopus, and Web of Science online databases for articles published between January 2000 and April 2023. The only inclusion criteria were (1) original research articles and studies on the application of AI-based medical clinical decision support using NLP techniques and (2) publications in English. A Critical Appraisal Skills Programme tool was used to assess the quality of the studies.

**Results:**

The search yielded 707 articles, from which 26 studies were included (24 original articles and 2 systematic reviews). Of the evaluated articles, 21 (81%) explained the use of NLP as a source of data collection, 18 (69%) used electronic health records as a data source, and a further 8 (31%) were based on clinical data. Only 5 (19%) of the articles showed the use of combined strategies for NLP to obtain clinical data. In total, 16 (62%) articles presented stand-alone data review algorithms. Other studies (n=9, 35%) showed that the clinical decision support system alternative was also a way of displaying the information obtained for immediate clinical use.

**Conclusions:**

The use of NLP engines can effectively improve clinical decision systems’ accuracy, while biphasic tools combining AI algorithms and human criteria may optimize clinical diagnosis and treatment flows.

**Trial Registration:**

PROSPERO CRD42022373386; https://www.crd.york.ac.uk/prospero/display_record.php?RecordID=373386

## Introduction

Advancement in medicine continues apace, especially with the emergence of new pathologies such as COVID-19. New treatments are continually being developed to fight not only these diseases but also previous pathologies for which new alternatives are being developed. Consequently, the number of publications in different indexed journals has increased, as shown in search results in various databases such as PubMed. Currently, it is possible to find many articles that mention new treatments or even new diagnostic forms [1,2].

Real and verified information is vital for the treatment and diagnosis of patients and is the cornerstone of medicine. The National Library of Medicine has developed at least 3 major source evaluation systems that provide useful examples for the task at hand: MEDLINE indexing, MedlinePlus indexing, and the Disaster Lit database [3].

Many health workers use the internet to search generally for updated clinical data [4]. However, this method is not the most efficient way to find information, since physicians must determine the type of information they need and then conduct the search themselves in an online medical database. This type of search can not only be time-consuming but also error prone due to not using suitable data. Therefore, automated information recommender systems have been established as a solution that allows medical staff to obtain reliable knowledge very quickly. These types of solutions are known as clinical decision support systems (CDSSs) [5].

CDSSs are composed of multiple platforms that allow the assessment of clinical data and alert clinicians to eventual problems. In addition, decision-making tools can be used to assist clinical staff. For these systems to function properly, they must interact with elements that allow them to obtain updated data for improved development, such as electronic health records (EHRs) [6]. Accordingly, CDSSs are known to focus on 6 specific aspects: data, knowledge, inference, architecture and technology, implementation and integration, and the user [7].

All available technology and tools (eg, artificial intelligence [AI], machine learning, and big data) could be useful for obtaining high-quality, reliable information. Such information could also be obtained by taking a supervised machine learning approach using several natural language processing (NLP) components that are domain independent and related to medical information extraction (text mining) [8]. These resources could include medical sources such as the Unified Medical Language System, different metathesauri, and different medical ontologies.

This study aims to answer the question of whether AI- and NLP-based CDSSs can provide effective results in automated searches that are useful to health care staff. To this end, a systematic review was carried out to assess the current state of these techniques in health care to identify their potential use in EHRs and automated information searches. The results found and conclusions drawn about the research question are subsequently presented.

## Methods

### Study Design

The protocol for this systematic review was published on November 5, 2022, in PROSPERO (CRD42022373386). This systematic review was performed per the PRISMA (Preferred Reporting Items for Systematic Reviews and Meta-Analyses) guidelines [9]. A search was conducted in the PubMed, Embase, ScienceDirect, Scopus, and Web of Science online databases for articles published between January 2000 and April 2023 using combinations of the following Medical Subject Headings (MeSH) terms: (((Artificial Intelligence [MeSH Terms]) AND (Natural language processing [MeSH Terms])) AND (Clinical decision support [MeSH Terms])) AND (Electronic health record [MeSH Terms]). The snowballing technique was used to complement the search to find the articles most relevant to the study [10].

### Selection Criteria

In total, 2 researchers independently assessed titles and abstracts and analyzed appropriate studies through full-text evaluation. The only inclusion criteria were (1) original research articles and studies on the application of AI-based medical clinical decision support (CDS) using NLP techniques and (2) publications in English. The exclusion criteria were (1) studies describing the use of AI that are not focused on CDS tools; (2) studies related only to NLP; (3) studies related to an algorithm submitted to a challenge; (4) letters to the editor; (5) conference abstracts, books and book reviews; and (6) studies not published in scientific journals (ie, only in science magazines or magazines without a DOI).

### Data Extraction and Management

Data were collected as follows: (1) reference, country, and year; (2) objective; (3) study type; (4) research design—intervention; (5) population sample + target (organ); and (6) results and conclusions. Further, 2 researchers independently extracted data. A third investigator resolved discrepancies.

### Quality Appraisal of the Studies

The articles were independently assessed by 2 researchers. Disagreements were discussed until a consensus was reached. A Critical Appraisal Skills Programme (CASP) tool for qualitative studies with a 10-item scale (0-10) [11] was used to ensure the quality of the studies, focusing on (1) validity of the study, (2) accuracy of the results, and (3) transferability. A 10-item CASP scale (0-10) was used for systematic reviews, focusing on (1) validity of the study, (2) robustness and relevance of the findings, and (3) applicability and relevance of the results in a local or specific context. Quality appraisal was used to demonstrate the methodological quality of the studies since it would affect the validity of the results and was something that needed to be taken into account when considering the findings of the review.

### Ethical Considerations

This study relied on secondary data. No ethics approval or patient consent was therefore required.

## Results

### Overview

**Figure 1:**
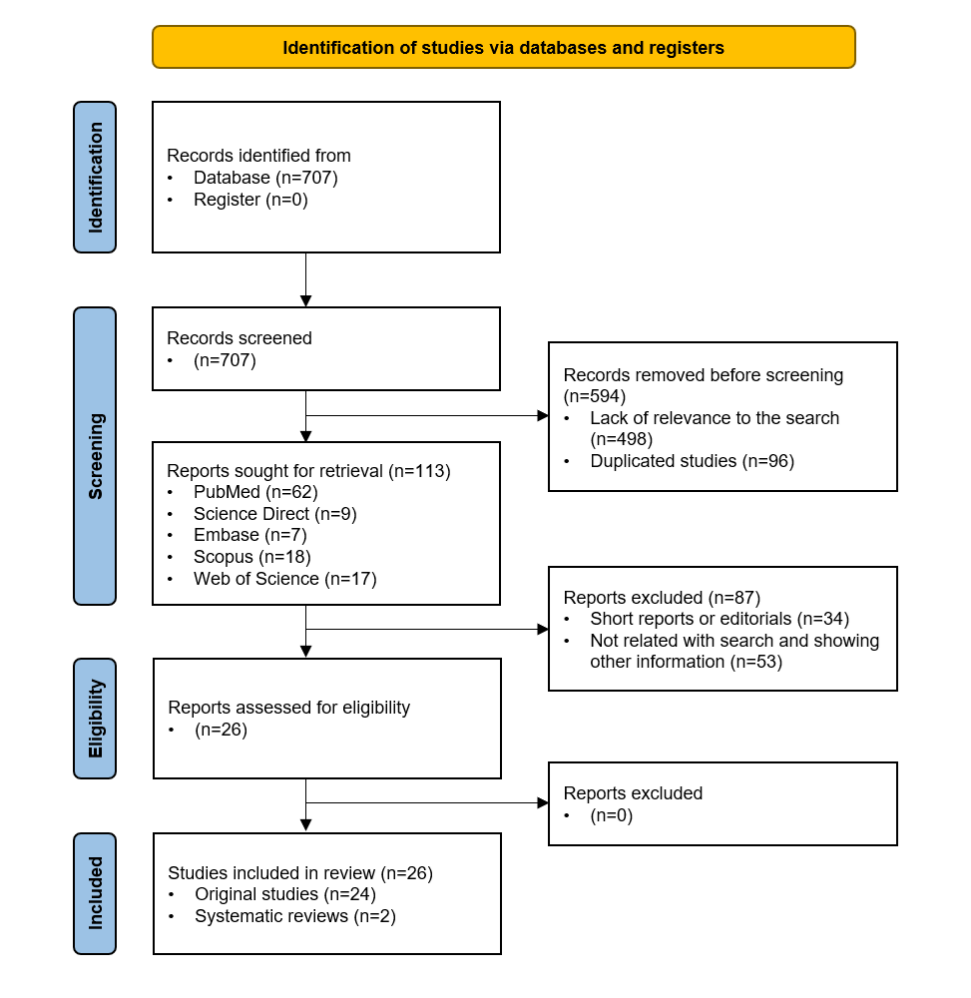
PRISMA 2020 flow diagram for new systematic reviews that include searches of databases and registers only. PRISMA: Preferred Reporting Items for Systematic Reviews and Meta-Analyses.

A systematic review was conducted with the aim of assessing the current state of AI and NLP techniques in health care to identify their potential use in EHRs and automated information searches. In the initial search, 707 articles were retrieved. In title and abstract screening, 594 publications were excluded either due to their lack of relevance to the search or duplication. After the initial review, 113 articles were chosen for further examination: 62 from PubMed, 9 from ScienceDirect, 7 from Embase, 18 from Scopus, and 17 from Web of Science. Of the remaining studies, 87 were excluded as they showed examples of data mining and algorithms for a challenge, presented nonscientific stories, or gave ultrashort presentations, among others. Therefore, 26 articles were included in the final analysis. The overview flowchart is shown in Figure 1.

### Characteristics of the Included Studies

The characteristics of the included studies are reported in Table 1 and Multimedia Appendix 1. In total, 24 of the evaluated articles were original articles and 2 were systematic reviews [12,13]. It should be noted that 6 of the original articles reviewed were presentations of studies conducted in relation to the 2010 i2b2/VA Workshop on Natural Language Processing Challenges for Clinical Records [8,14-18].

**Table 1:** Summary of the studies included in the systematic review (n=26).

Study	Objective	Research design—intervention	Population sample + target (organ)	Results and conclusions
Clark et al [14]	To develop a system for determining the assertion status of clinical reports (extracted from patient records)	Determining what kind of clinical assertion was made in a clinical record (problem concept-sorted). 3 assertions: conditional hypothetical and not associated with the patient	2010 i2b2^a^/VA^b^: 349 clinical records. Detection of negation, speculation, and conditionals	Linguistic information (negation status and temporal attributes) are features that are not always evaluated. They could give a more accurate classification
Patrick et al [15]	To present a method to deal with different extractions of, and classifications in, clinical data	Developing clinical NLP^c^ that includes a proofreading process with validation and correction	2010 i2b2/VA: 349 manually annotated notes + 827 raw records: 479 records	NLP in clinical fields needs to address the issues between model complexity and model accuracy
Roberts and Harabagiu [8]	To develop a framework that can optimally identify medical concepts and adequately classify assertions	Evaluating and finding the near-optimal subset of features for the 2010 i2b2/VA context task (concept extraction)	2010 i2b2/VA: 827 summaries	Machine-learning–based supervised methods can be improved with feature-selection techniques
Jiang et al [16]	To design and evaluate a machine-learning algorithm to extract clinical information from hospital discharge summaries	Evaluating the effects of different types of features	2010 i2b2/VA: 349 clinical annotated notes	A system that is useful for concept extraction and assertion
D’Avolio et al [17]	To evaluate if the use of NLP-derived features combined with supervised machine-learning can perform effectively across tasks	Creating an engine using cTAKES^d^ (“noun-phrase bumpers”) for text extraction with NLP combined with machine-learning classification	2010 i2b2/VA: 349 clinical annotated notes	NLP clinical extraction is well known. Its adoption with acceptable concept-level extraction performance is also known
Garla et al [19]	To develop a classifier extractor using cTAKES to store documents	Exploring if the accuracy of rule-based classifiers could be improved with machine-learning algorithms (testing systems after improvement)	13,000 abdominal radiology reports, ascites, and liver masses of 395 patients	cTAKES extensions simplify feature extraction—it enables a better and more efficient exploration of text including the syntactic structure of documents and the negation context of concepts
Wagholikar et al [20]	To develop a CDSS for cervical cancer screening that can interpret free text Papanicolaou reports	Developing a CDSS^e^ that would be able to identify abnormal Papanicolaou reports as well as interpret other variables accurately	74 patient cases from cervical cancer screening	EHR^f^ text can be effectively used through NLP to develop CDS^g^ tools.
Wagholikar et al [21]	To evaluate a CDSS for cervical cancer screening	A previous CDSS was created, care providers’ recommendations were requested and compared with the CDSS system	6053 random patients and 8 reviewers. Cervical cancer screening	CDS and human review can facilitate the evaluation of accuracy and would help to identify decision scenarios that may be missed
Sordo et al [22]	To develop a conceptual schema to represent clinical knowledge for decision support	Rule interactions based on semantic web technologies (ontologies). Evaluating the feasibility of the modelling strategy by implementing test scenarios	3 applications were analyzed: laboratory results; adverse drug reaction monitoring and immunization protocols	A richer conceptual representation of production rules will facilitate rule authoring consistency in rule implementation and maintenance. Interactions are not considered a modelling time but they should be
Mehrabi et al [23]	To develop an NLP system to identify (retrospective) patients with pancreatic cysts	Records were evaluated by the system created. This system double checks negation in the EHR and stratifies the results (low or high risk of cancer)	Test set of 703 patient records: pancreas and cyst. Training set: 316 control records	Identification of negation improved the algorithm, increasing recall to 98.12%
Ross et al [12]	To use secondary data and to put data into practice	Paper-based materials containing similar information. Finding examples of BD^h^ use with EHRs	84 papers—EHR research (various organs)	Greater use of EHRs means more clinical data. There is a need to develop new methods to obtain better data from EHRs
Kotfila and Uzuner [18]	To evaluate if NLP techniques can identify phenotypes in unstructured medical notes	Training support vector machines using individual feature spaces to assess better model performance	2 separate i2b2 shared tasks (2008+2014: 790+730 documents)	A simple feature performed as well as a combination of feature spaces—the addition of training data has weak statistical significance effects
Patterson et al [24]	To determine whether a colonoscopy was performed for screening	Creating a rule-based model and 3 machine-learning models (automated retrieval console) which also uses cTAKES	A set of 1000 documents with colonoscopies	A rule-based classification system appears to be more robust than a machine-learning system alone
Divita et al [25]	To use an NLP algorithm to process large corpora of clinical notes to demonstrate a time decrease in the analyses of a large corpus of clinical information	Developing an NLP algorithm to detect urinary catheters in hospitalized patients	550,000 notes from patients with urinary catheters from a US Veteran Affairs hospital	Generalizable and widely applicable techniques can aid BD processing and analysis
Mei et al [26]	To evaluate a decision fusion framework for treatment recommendation systems (combining knowledge-driven and data-driven decisions)	Implementing a fusion engine that obtains inputs from base decision engines	3150 records from patients with diabetes in China	Decision fusion is a promising way toward a more valuable treatment recommendation
Marco-Ruiz et al [27]	To find out if multidisciplinary leverage archetypes and ontologies can model CDSS (better reuse and maintenance)	An archetype was developed and reviewed by 5 practitioners and 5 information architects. SNOMED for concept simplification	126 cases with respirator and symptoms and signs	Model archetypes can provide information using ontologies. EHR standards and terminologies can be used by multidisciplinary teams effectively
Danger et al [28]	To explain the methodology for constructing a clinical prediction rule repository	Using data mining algorithms for pattern extraction	Clinical collection for 126,931 patients from primary care with multiple diagnoses	Important factors to be included in the rules were defined. With the use of clinical evidence rules, a better diagnostic recommendation can be obtained
Breischneider et al [29]	To introduce a system for the automated processing of clinical reports of patients with mamma carcinoma to extract relevant textual features and derive therapy suggestions	Use of a rule-based grammar approach for information extraction and deriving therapy suggestions based on the extracted variables and clinical guidelines. The accuracy of predicting therapeutic measures is also reported	8766 clinical text reporting on 2096 patients with mamma carcinoma	Good accuracy of information extraction for different features. The evaluation of therapy suggestions, factors influencing the error between suggested and conducted therapy
Yang et al [30]	To use convolutional neural networks to be able to potentiate extraction without paper construction of rules or knowledge bases	Proposing a method using neural networks to extract features from EHRs. A tool was used in medical text showing 98.7% accuracy and 96.02% recall	18,590 EHRs indicating hypertension, diabetes, COPD^i^, gout, arrhythmia, asthma, gastritis, and stomach polyps. After validation: a training set of 7000 EHRs and a test set of 400 EHRs	The use of neural networks can increase information extraction. This could assist in a feasible and effective diagnosis
Wissel et al [31]	To validate an NLP application using machine-learning to identify patients for epilepsy surgery	Determining the proportion of potential surgical candidates using EHRs, NLP, and a surgical score	1 app and 4211 patients with an epilepsy diagnosis after surgical treatment of epilepsy and controls	NLP-based app modelled for patient score to identify candidates for epilepsy surgery
Wulff et al [32]	To design a tool to automatically extract important information from medical texts and transform them into standardized data	NLP consisting of 5 successive tasks was developed. The tool was implemented to extract fragments from EHRs (97% precision and 94% recall)	529 medical events from the pediatric cardiology and the intensive care medicine department at Hannover Medical School. Further, 499 events were correctly extracted	NLP-based solution to extract information from medical text. This will benefit health care by analyzing a large amount of information and presenting the most important data
Kulchak et al [33]	To evaluate if modification of a CDS tool using concepts of human-centered design can improve CDS itself	Adapting a DDSS^j^ (design, NLP with cTAKES, and coordination with EHRs). Creating simulated cases using real but deidentified clinical information (varying complexity)	3 simulated genetic cases of increasing complexity were created, 3 clinicians tested the system and another 3 retested it	Evidence of potential implementation of DDSS using machine-assisted medical record review. The use of mixed technical CDS and human-centered design criteria enhances the real-world clinical workflow
van de Burgt et al [13]	To assess whether data mining can improve the diagnostic and therapeutic processes of CDS	Combining data mining and CDSSs may improve diagnostic and therapeutic processes, contributing to increased patient safety	714 reviewed publications, 39 of which were included	Barriers: complexity of NLP, EHR incompleteness, validation and performance of the tool, lack of input from an expert team, and the adoption rate among professionals
Suh et al [34]	To evaluate the use of clinical NLP to identify elements relevant to preoperative medical history by analyzing clinical notes	An NLP pipeline would identify a significant portion of pertinent history captured by a perioperative provider (in an EHR)	93 patients with 9765 clinical notes were reviewed	The use of NLP identified medical conditions relevant to preanesthetic evaluation from unstructured free-text input. This provides CDS or recommends additional testing or evaluation
Park et al [35]	To evaluate the efficacy of an algorithm of 3 levels of search functionality in supporting information retrieval for clinical users from EHR in a simulated clinical environment	Medical tasks were presented to an evaluation system and a panel of medical raters to assess both the speed and accuracy of task completion	Medical records for 3 fictional patients, each with 20 documents, were assessed by 60 clinicians of varying specialties and experience levels	NLP-enhanced search facilitated more accurate task completion than both string search and no search, with similar task speeds between NLP-enhanced search and string search
Afshar et al [36]	To implement a real-time NLP-driven CDS tool for screening opioid misuse in hospitalized adults and assess its effectiveness in providing interventions for substance use disorder treatment	A 30-month quasi-experimental pre-post study screened inpatients for 24 months of standard care followed by a 6-month tool implementation phase. Cost-effectiveness analysis and user acceptability evaluations were covered	A total sample size of 12,500 patients (10,000 preintervention and 2500 postintervention)	The study implemented a real-time NLP-driven CDS tool for screening opioid misuse with a sensitivity of 93% and specificity of 92%. The workflow was reproducible and included a shared pseudocode for cloud service implementation

^a^i2b2: Informatics for Integrating Biology and the Bedside.

^b^VA: US Department of Veterans Affairs.

^c^NLP: natural language processing.

^d^cTAKES: Clinical Text Analysis and Knowledge Extraction System.

^e^CDSS: clinical decision support system.

^f^EHR: electronic health record.

^g^CDS: clinical decision support.

^h^BD: big data.

^i^COPD: chronic obstructive pulmonary disease.

^j^DDSS: diagnostic decision support system.

Of the 26 articles reviewed, 18 (69%) corresponded to authors from the United States [8,12-14,16-25,31,33,34,36]; China [26,30], Germany [29,32], the United Kingdom [28,35] each had 2 (8) articles; and the rest (n=2, 8%) were from Norway [27] and Australia [15].

Of the evaluated articles, 21 (81%) explained the use of NLP as a source for data collection, and 18 (69%) articles used EHRs as a data source; meanwhile, a further 8 (31%) articles were based on clinical data [8,12,14-16,18-21,23-25,29-33,36]. Only 5 (19%) articles showed the use of an NLP tool called Apache cTAKES (Clinical Text Analysis and Knowledge Extraction System) as a set of combined strategies for NLP to obtain clinical data [14,16,17,19,36].

A total of 16 (62%) articles presented stand-alone data review algorithms [20-33,35,36]. Other studies (n=9, 35%) showed that the CDSS alternative was also a way of displaying the information obtained for immediate clinical use [12,20,22,26-28,30,33,36].

Some of the articles focused on specific pathologies such as epilepsy [31]; genomics [33]; pancreatic cysts [23]; radiographic images [19]; diabetes, obesity, hypertension, dyslipidemia, and cardiovascular diseases [18]; colonoscopies [24]; urinary problems or catheters [25]; posttraumatic stress disorder [17]; preanesthetic evaluation [34]; breast cancer [29]; opioid misuse [36]; and cervical cancer assessment [20,21], thus emphasizing the use that can be made of these types of system in almost all medical specialties. In addition, it should be noted that the vast majority of studies reviewed did not consist of just a few cases. This is demonstrated by the fact that 74 patients and their EHRs were reviewed in 1 study [20]; 349 clinical cases were reviewed in several studies [8,14-18]; and more than 126,000 clinical cases were analyzed by information collection systems in another [28].

### Quality Appraisal Results

In the CASP checklist for the 24 qualitative studies (Multimedia Appendix 2), all had a clear statement of the aims of the research, an adequate qualitative study design, and clearly defined outcomes. Data collection and analysis were sufficiently rigorous in the 24 studies, all were adequately designed to achieve the research aims, and the results obtained were readily transferrable to other settings. However, only 4 of the evaluated studies [20,21,27,31] indicated interaction with the participants (patients, in this instance), which involved informing them about the study and the use of the data obtained from it. A further 12 studies [18,19,23-25,28-30,32-34,36] also used patient data or health records, but there was no mention of patients being informed. Importantly, while the remaining 7 studies used data for their research, 5 [8,15-17,37] obtained the necessary information from a database for a challenge (i2b2/VA), 1 study generated clinical data specifically for the research [35], and the other 2 [22,26] did not mention the type of data they used or where they obtained it from. Nevertheless, none of this influenced or affected the results of the research. All the studies were analyzed using standard means of content analysis and provided sufficient information on the design to replicate the study. This was sufficient to demonstrate the credibility of the studies and that the data analysis was sufficiently rigorous.

In the CASP checklist for 2 systematic reviews (Multimedia Appendix 2), both studies had a clear statement of research objectives, an appropriate study design, clearly defined outcomes, and sufficiently rigorous data collection and analysis. Both studies were adequately designed to achieve the research aims, and the results obtained were readily transferable to other settings.

## Discussion

### Principal Findings

This systematic review produced a synthesis of the current state of AI and NLP techniques in health care to identify their potential use in EHRs and automated information searches. Most of the studies showed good internal validity and decent quality. What stands out from our study is the use of NLP as a source for data collection, and while most of the included studies used EHRs, some were based on clinical data. Only 5 of the articles indicated the use of combined NLP strategies to obtain clinical data. While more than half (16/26, 62%) of the articles presented stand-alone algorithms for data review, others (9/26, 35%) indicated that CDSSs also served to present the information obtained for immediate clinical use.

NLP, as a data mining technique, is considered one of the most appropriate tools to find useful information in the data contained in large databases [12,16]. This is because it is an instrument that enables large amounts of information to be clinically analyzed, showing only the parts with the greatest interest or importance to health professionals [32]. While its use has significantly advanced in extracting concepts from clinical data [17], it faces challenges when dealing with the unstructured format of EHRs, which can impede accurate responses to queries submitted to NLP [15].

To overcome these challenges, various techniques have been proposed. One approach involves the combined use of clinical scores that serve as a guide for obtaining results [31], which could be very useful in improving health systems. Another technique to enhance data collection could be the use of neural networks to increase information extraction (and thus achieve more effective diagnoses) [30]. An alternative option offered by NLP includes a sentiment-based model that goes beyond the traditional collaborative filtering approach. This model uses machine learning algorithms to analyze human language text. The metrics used in sentiment analysis aim to determine whether the overall tone of a text is positive, negative, or neutral [38].

Algorithms are the basis of NLP, which consist of any well-defined computational procedure that takes a value or set of values as input and produces a value or set of values as output [39]. However, despite algorithms being versatile tools used in programming and software development, and predominantly acknowledged for their pivotal role in data mining and AI [40], the process of algorithm development is not always straightforward and can sometimes become complicated. For example, many algorithms have difficulties with “negation,” as it can be interpreted as a positive part of a patient’s clinical history (thus “does not smoke” can be understood as “smoker”). This is a linguistic problem with features that are not always valued, which can lead to inaccurate classification [14,23,41]. It is for this reason that solutions such as NegEx, an algorithm developed in 2001, have been created to try to correct the problem with negation [42]. It should be noted that the use of rules (heuristics) in the search for clinical evidence can generate a better diagnostic recommendation [28], and this is probably because classification systems using rules present more robust machine learning models [24]. The use of tools such as cTAKES is also an alternative, as they are more efficiently and accurately able to scan texts and even the syntactic structure of documents, including negation [19].

The results show the potential of using NLP not only in reviewing clinical notes but also with algorithms that can help find specific information in large volumes of medical information [25]. This may explain its widespread use in epidemiology, public health, and disease surveillance [43,44]. The data obtained could be used to prevent new outbreaks of different diseases worldwide and to identify the main characteristics of pathologies to guide diagnoses even before the disease develops to chronic levels. Health care professionals could benefit from integrating NLP with AI in CDSS to improve medical consultations, streamline tasks such as data analysis, document clinical information in an automated and structured way, and refine treatment strategies and diagnostic processes by automated identification and extraction of key data from medical records [29]. Providing accurate information in real time could improve medical decision-making that better suits each patient’s individual needs, which could translate into better medical outcomes.

When grouping the results by their findings, several conclusions could be drawn, such as that NLP is effective in a CDSS, very accurate, and faster than manual search, especially when accompanied by a human review to facilitate the evaluation of the results and check their accuracy. However, it is necessary to consider the fact that more clinical data from EHRs may complicate its use and that new methods would have to be developed to better obtain large amounts of data. Another striking aspect is that all the reviewed articles focus on the detection of clinical data in EHRs in closed environments. That is, the information obtained was used to account for specific pathologies or diagnostic procedures, and the accuracy was assessed by someone able to understand EHRs. However, none of the articles reviewed referred to the use of external data (medical databases); they all use the data found—using NLP—in the EHRs only. By using external data sources, more appropriate or updated diagnostic aids and treatments could be obtained.

There are also some barriers preventing the development and improvement of NLP systems. One such barrier is the lack of data or incomplete data in EHRs. Another is associated with the lack of use or knowledge of NLP by health professionals. The latter significant issue is the lack of multidisciplinary working practices (health care and computer specialists), which hinders adequate progress concerning NLP algorithms. Establishing a multidisciplinary team involving physicians and information systems professionals would be the most effective approach, as demonstrated in various health care environments [45-47]. In total, 6 of the reviewed articles described the results of a challenge to find an algorithm that best uses NLP in clinical notes, underscoring the efficacy of such initiatives in catalyzing technological advancement, thereby enhancing the performance of algorithms applicable in AI and big data domains.

Incorporating several medical ontologies to increase the coverage of medical entities may enhance results [48]. A semantic term, representing a single clinical concept, serves as a starting point for ontologies. The combination of these concepts defines a set of properties, allowing interconnections (mapping) between them. This process generates semantic ontologies, characterized by controlled terminology and formal semantic relationships in a particular area of interest using a particular modelling language and terminology [22], such as the terms in EHRs [27]. The incorporation of machine learning techniques into EHRs not only produces better results but also plays a key role in the development of predictive rules. Through the use of ontologies, diagnoses are standardized with a unified vocabulary, facilitating seamless exchange and validation across diverse populations [18,28].

The weight given to ontologies in the studies reviewed varies. While some of them define their use very well [22,27], others only mention ontologies as an important part of information extraction [8,12,15,17-19,23,28,29,33,34] or not at all [13,14,16,21,24-26,30-32,35,36]. Further, 1 study mentions the word “ontology” in the keywords but not in the text. This is surprising, as health ontologies are a fundamental part of clinical data extraction projects, and even more so considering the emergence of new ontologies with almost every new study. The levels of understanding of ontology concepts where the knowledge domains of medicine and computer science intersect could be reviewed as a future line of research.

Regarding the use of ontologies, their inclusion with the use of the semantic web, along with medical NLP, will lead to a better assessment of annotation tasks [49,50]. The use of ontologies is extremely important to overcome the barriers that may arise with the use of NLP. For example, to overcome them, some proposals could be adopted, such as the use of (1) AI assistants (special fusion engines) combining knowledge-based engines and data-based engines; (2) biphasic tools (adding human intervention) with the addition of a human reviewer, which would improve search results and identify potentially lost data; and (3) semantic graphs (sentiment analysis), where ontology-based AI tools would allow relevant information about pathologies in clinical data to be found.

The use of appropriate ontologies in NLP systems would serve to facilitate the real-time extraction of information that could be used for the development of real-time clinical decision tools [51,52]. Ontologies can also be useful for avoiding the ambiguity and inconsistencies found in some health care documents such as EHRs [53]. This is very important because these clinical documents could be converted into more understandable semantic structures by the NLP algorithm, allowing the most important information to be extracted [54]. Thus, a CDSS with incorporated NLP could provide physicians with contextual information, meaning that better clinical decisions could be made to the benefit of patients [55]. Such improvements could take the form of system-generated alerts when alterations in vital sign monitoring or interactions between prescribed drugs are detected [56-58].

Although CDSSs have great potential for use by health care staff to increase adherence to clinical guidelines and to assist in the correct diagnosis, treatment, follow-up, and prevention of various pathologies, with the consequent better maintenance of the population’s health [7], some studies suggest that they may disrupt physicians’ workflow or alter or be inconsistent with the initial clinical decisions, and may also require technical maintenance with additional costs [59]. Thus, depending on the algorithm and validation, it may present incorrect or low-quality data. Furthermore, as a different program from the EHR one, there may not be adequate interoperability between the two.

With this information, the advantages of using NLP and CDSSs are obvious. However, it is noteworthy that all the studies in our review have a “closed behavior,” meaning that only specific information is searched for in the data present in clinical notes, without searching for further information in the large medical databases available. If the latter were to be carried out, it would allow a new line of research to be developed, in which NLP-based algorithms combined with keyword searches in clinical databases such as PubMed could potentially enable better and faster diagnoses to be made, and also updated treatments to be offered, all based on EHR data and in real time.

The ongoing evolution of generative AI, namely large language models (LLMs), represents a type of AI that is capable of generating text through a process of training on large data sets in multiple languages. These models demonstrate the ability to produce “human-like” responses [60]. A well-known example is ChatGPT, whose architecture uses a neural network to process natural language, thus generating responses based on the context of the input text [61]. It is essential to recognize that the synergistic use of these techniques presents a significant opportunity. The integration of tools based on LLM, medical ontologies, and NLP has the potential to offer a substantial positive influence on the health care process [62].

These results support the need to conduct research aimed not only at improving algorithms and generating new knowledge but also at suggesting new research directions for the development of AI tools. This includes the integration of NLP, medical ontologies, and LLM for enhanced search capabilities in EHRs and other external sources. A promising research path could be to develop algorithms whose architecture is based on web systems and contrasted medical databases, supported by AI with NLP, and that gather information about semantic terms from health care ontologies such as those in the National Library of Medicine. Such developments of AI-based tools may have a positive impact on research into their use in certain areas, such as health care [63]. In addition, the development of AI-based skills also enhances the development of further algorithms and research, as evidenced by the publications resulting from the challenges mentioned above. However, it is imperative to acknowledge the potential ethical implications inherent in this field, which require thorough assessment and subsequent integration into clinical practice [64]. While the potential benefits are substantial, it is paramount to rigorously address ethical considerations and data privacy concerns, emphasizing cybersecurity and privacy requirements to effectively protect patients’ sensitive data and ensure their confidentiality [36,65,66].

### Limitations

Despite conducting an exhaustive search across 5 databases, which specifically targeted studies on the application of AI-based medical CDS using NLP techniques, a total of 113 studies were initially identified for screening. However, upon thorough review, only 26 studies were deemed to meet the stringent inclusion and exclusion criteria established for this review. Consequently, the representativeness of our findings may be questioned given the number of records primarily identified and the possible paucity of research on this particular study topic. A significant number of articles were excluded from our review due to their failure to establish a clear connection between NLP, AI, medical records, and their integration with CDSS. Despite delving into NLP and AI within the context of medical records, these articles lacked sufficient exploration of their relationship with CDSS [67]. The sources of information were peer-reviewed publications, so relevant information from other sources (eg, gray literature) was omitted. CASP-based quality scores [11] may have reflected incomplete reporting, since the vast majority of studies did not compare their results to those of other studies along similar lines (eg, the 2010 i2b2/VA challenge), or had short lists of references (between 8 and 20) in which nonscientific ones were included [14,22,23,25-28]. Nevertheless, all the articles were very robust in terms of the presentation of their results, which could be extrapolated to different local communities without losing their essence.

### Conclusions

The use of NLP engines can effectively obtain results that guide the development of more accurate clinical decision systems. The implementation of decision systems using AI assistants is a potential use of this type of tool. Furthermore, the use of biphasic tools using AI criteria as algorithms combined with human criteria may improve the flow of clinical diagnosis or treatment. Human review can improve the accuracy of the search results as well as identify scenarios that might have been missed. The implementation of a special fusion engine (combining knowledge-driven and data-driven engines) is a promising technique that has shown results in terms of more relevant (or improved) recommendations.

Most CDSSs are designed to recommend text based on keywords. However, this leads to problems regarding the effectiveness of the method using NLP. Some proposals, such as the use of semantic graphs, have been put forward to solve this problem. Some controversy has arisen over the fact that CDSSs endure problems related to a certain coldness in their responses, as well as a paucity of data. A sentiment analysis technique to evaluate user preferences may help to overcome this.

The results found allow us to establish new lines of research for the development of AI tools based on NLP with the use of medical ontologies for information searching in both EHRs and external sources (clinical databases) to obtain better results and extra information that could be used to the benefit of patients.

## Appendix

Multimedia Appendix 1 Main characteristics of the studies included in the systematic review.

Multimedia Appendix 2 Quality appraisal—CASP checklist for qualitative studies and for systematic review. CASP: Critical Appraisal Skills Programme.

Multimedia Appendix 3 PRISMA 2020 checklist. PRISMA: Preferred Reporting Items for Systematic Reviews and Meta-Analyses.

## Abbreviations

AI: artificial intelligence
CASP: Critical Appraisal Skills Programme
CDS: clinical decision support
CDSS: clinical decision support system
cTAKES: Clinical Text Analysis and Knowledge Extraction System
EHR: electronic health record
LLM: large language model
MeSH: Medical Subject Headings
NLP: natural language processing
PRISMA: Preferred Reporting Items for Systematic Reviews and Meta-Analyses
