# Supplementary material for: Clinical Decision Support and Natural Language Processing in Medicine: Systematic Literature Review
Source: J Med Internet Res. 2024 Sep 30;26:e55315. doi: 10.2196/55315 (PMC11474138; doi:10.2196/55315)
Supplement: Multimedia Appendix 1 [file jmir_v26i1e55315_app1.docx]

**Table S1.** Main characteristics of the studies included in the systematic review (n=26).

| **Review ID** | **Country/Year** | **Study type** | **Quality (CASP)** |
| --- | --- | --- | --- |
| [14] | USA  (2011) | Original | 9/10 |
| [15] | Australia  (2011) | Original | 9/10 |
| [8] | USA  (2011) | Original | 9/10 |
| [16] | USA  (2011) | Original | 9/10 |
| [17] | USA  (2011) | Original | 9/10 |
| [20] | USA  (2011) | Original | 8/10 |
| [23] | USA  (2012) | Original | 10/10 |
| [19] | USA  (2013) | Original | 10/10 |
| [24] | USA  (2013) | Original | 6/10 |
| [27] | USA  (2013) | Original | 8/10 |
| [12] | USA  (2014) | Review | 10/10 |
| [18] | USA  (2015) | Original | 9/10 |
| [21] | USA  (2015) | Original | 8/10 |
| [22] | USA  (2015) | Original | 8/10 |
| [30] | China  (2015) | Original | 8/10 |
| [36] | Norway  (2015) | Original | 9/10 |
| [34] | United Kingdom  (2015) | Original | 8/10 |
| [33] | Germany  (2017) | Original | 8/10 |
| [31] | China  (2018) | Original | 8/10 |
| [26] | USA  (2020) | Original | 10/10 |
| [32] | Germany  (2020) | Original | 8/10 |
| [25] | USA  (2021) | Original | 9/10 |
| [13] | USA  (2022) | Review | 10/10 |
| [28] | USA  (2022) | Original | 8/10 |
| [35] | United Kingdom  (2022) | Original | 8/10 |
| [29] | USA  (2023) | Original | 9/10 |

**References**

8. Roberts K, Harabagiu SM. A flexible framework for deriving assertions from electronic medical records. J Am Med Inform Assoc. 2011;18: 568. doi:10.1136/AMIAJNL-2011-000152

12. Ross MK, Wei W, Ohno-Machado L. “Big Data” and the Electronic Health Record. 2014. doi:10.15265/IY-2014-0003

13. van de Burgt BWM, Wasylewicz ATM, Dullemond B, Grouls RJE, Egberts TCG, Bouwman A, et al. Combining text mining with clinical decision support in clinical practice: a scoping review. Journal of the American Medical Informatics Association. 2023;30: 588–603. doi:10.1093/JAMIA/OCAC240

14. Clark C, Aberdeen J, Coarr M, Tresner-Kirsch D, Wellner B, Yeh A, et al. MITRE system for clinical assertion status classification. J Am Med Inform Assoc. 2011;18: 563. doi:10.1136/AMIAJNL-2011-000164

15. Patrick JD, Nguyen DHM, Wang Y, Li M. A knowledge discovery and reuse pipeline for information extraction in clinical notes. J Am Med Inform Assoc. 2011;18: 574. doi:10.1136/AMIAJNL-2011-000302

16. Jiang M, Chen Y, Liu M, Rosenbloom ST, Mani S, Denny JC, et al. A study of machine-learning-based approaches to extract clinical entities and their assertions from discharge summaries. J Am Med Inform Assoc. 2011;18: 601. doi:10.1136/AMIAJNL-2011-000163

17. D’Avolio LW, Nguyen TM, Goryachev S, Fiore LD. Automated concept-level information extraction to reduce the need for custom software and rules development. J Am Med Inform Assoc. 2011;18: 607. doi:10.1136/AMIAJNL-2011-000183

18. Kotfila C, Uzuner Ö. A systematic comparison of feature space effects on disease classifier performance for phenotype identification of five diseases. J Biomed Inform. 2015;58: S92. doi:10.1016/J.JBI.2015.07.016

19. Wagholikar Dr. KB, MacLaughlin KL, Kastner TM, Casey PM, Henry M, Greenes RA, et al. Research and applications: Formative evaluation of the accuracy of a clinical decision support system for cervical cancer screening. J Am Med Inform Assoc. 2013;20: 749. doi:10.1136/AMIAJNL-2013-001613

20. Garla V, Re V Lo, Dorey-Stein Z, Kidwai F, Scotch M, Womack J, et al. The Yale cTAKES extensions for document classification: architecture and application. J Am Med Inform Assoc. 2011;18: 614. doi:10.1136/AMIAJNL-2011-000093

21. Patterson O V., Forbush TB, Saini SD, Moser SE, Duvall SL. Classifying the Indication for Colonoscopy Procedures: A Comparison of NLP Approaches in a Diverse National Healthcare System. Stud Health Technol Inform. 2015;216: 614–618. doi:10.3233/978-1-61499-564-7-614

22. Divita G, Carter M, Redd A, Zeng Q, Gupta K, Trautner B, et al. Scaling-up NLP pipelines to process large corpora of clinical notes. Methods Inf Med. 2015;54: 548–552. doi:10.3414/ME14-02-0018/ID/JR0018-16

23. Wagholikar KB, MacLaughlin KL, Henry MR, Greenes RA, Hankey RA, Liu H, et al. Clinical decision support with automated text processing for cervical cancer screening. J Am Med Inform Assoc. 2012;19: 833. doi:10.1136/AMIAJNL-2012-000820

24. Sordo M, Rocha BH, Morales AA, Maviglia SM, Dell’Oglio E, Fairbanks A, et al. Modeling Decision Support Rule Interactions in a Clinical Setting. Stud Health Technol Inform. 2013;192: 908–912. doi:10.3233/978-1-61499-289-9-908

25. Kulchak Rahm A, Walton NA, Feldman LK, Jenkins C, Jenkins T, Person TN, et al. User testing of a diagnostic decision support system with machine-assisted chart review to facilitate clinical genomic diagnosis. BMJ Health Care Inform. 2021;28. doi:10.1136/BMJHCI-2021-100331

26. Wissel BD, Greiner HM, Glauser TA, Holland-Bouley KD, Mangano FT, Santel D, et al. Prospective Validation of a Machine Learning Model that Uses Provider Notes to Identify Candidates for Resective Epilepsy Surgery. Epilepsia. 2020;61: 39. doi:10.1111/EPI.16398

27. Mehrabi S, Schmidt CM, Waters JA, Beesley C, Krishnan A, Kesterson J, et al. An Efficient Pancreatic Cyst Identification Methodology Using Natural Language Processing. Stud Health Technol Inform. 2013;192: 822–826. doi:10.3233/978-1-61499-289-9-822

28. Suh HS, Tully JL, Meineke MN, Waterman RS, Gabriel RA. Identification of Preanesthetic History Elements by a Natural Language Processing Engine. Anesth Analg. 2022;135: 1162–1171. doi:10.1213/ANE.0000000000006152

29. Afshar M, Adelaine S, Resnik F, Mundt MP, Long J, Leaf M, et al. Deployment of Real-time Natural Language Processing and Deep Learning Clinical Decision Support in the Electronic Health Record: Pipeline Implementation for an Opioid Misuse Screener in Hospitalized Adults. JMIR Med Inform. 2023;11: e44977. doi:10.2196/44977

30. Mei J, Liu H, Li X, Xie G, Yu Y. A Decision Fusion Framework for Treatment Recommendation Systems. Stud Health Technol Inform. 2015;216: 300–304. doi:10.3233/978-1-61499-564-7-300

31. Yang Z, Huang Y, Jiang Y, Sun Y, Zhang Y-J, Luo P. Clinical Assistant Diagnosis for Electronic Medical Record Based on Convolutional Neural Network. ScIeNTIfIc REPORtS |. 2018;8: 6329. doi:10.1038/s41598-018-24389-w

32. Wulff A, Mast M, Hassler M, Montag S, Marschollek M, Jack T. Designing an openEHR-Based Pipeline for Extracting and Standardizing Unstructured Clinical Data Using Natural Language Processing. Methods Inf Med. 2020;59: e64. doi:10.1055/S-0040-1716403

33. Breischneider C, Zillner S, Hammon M, Gass P, Sonntag D. Automatic Extraction of Breast Cancer Information from Clinical Reports. Proc IEEE Symp Comput Based Med Syst. 2017;2017-June: 213–218. doi:10.1109/CBMS.2017.138

34. Danger R, Corrigan D, Soler JK, Kazienko P, Kajdanowicz T, Majeed A, et al. A methodology for mining clinical data: experiences from TRANSFoRm project. Stud Health Technol Inform. 2015;210: 85–89. doi:10.3233/978-1-61499-512-8-85

35. Park EH, Watson HI, Mehendale F V, O’Neil AQ, Clinical Evaluators. Evaluating the Impact on Clinical Task Efficiency of a Natural Language Processing Algorithm for Searching Medical Documents: Prospective Crossover Study. JMIR Med Inform. 2022;10: e39616. doi:10.2196/39616

36. Marco-Ruiz L, Maldonado JA, Karlsen R, Bellika JG. Multidisciplinary Modelling of Symptoms and Signs with Archetypes and SNOMED-CT for Clinical Decision Support. Stud Health Technol Inform. 2015;210: 125–129. doi:10.3233/978-1-61499-512-8-125
