# Supplementary material for: Clinical Decision Support and Natural Language Processing in Medicine: Systematic Literature Review
Source: J Med Internet Res. 2024 Sep 30;26:e55315. doi: 10.2196/55315 (PMC11474138; doi:10.2196/55315)
Supplement: Multimedia Appendix 2 [file jmir_v26i1e55315_app2.docx]

Quality Appraisal – CASP Checklist for Qualitative Studies

|  | **1** | **2** | **3** | **4** | **5** | **6** | **7** | **8** | **9** | **10** | **Quality rating** |
| --- | --- | --- | --- | --- | --- | --- | --- | --- | --- | --- | --- |
| Clark et col [1] | Y | Y | Y | Y | Y | Can’t Tell | Y | Y | Y | Y | **9** |
| Patrick JD et col [2] | Y | Y | Y | Y | Y | Can’t Tell | Y | Y | Y | Y | **9** |
| Robert K et col [3] | Y | Y | Y | Y | Y | Can’t Tell | Y | Y | Y | Y | **9** |
| Jiang M et col [4] | Y | Y | Y | Y | Y | Can’t Tell | Y | Y | Y | Y | **9** |
| D Avolio LW et col [5] | Y | Y | Y | Y | Y | Can’t Tell | Y | Y | Y | Y | **9** |
| Garla V et col [6] | Y | Y | Y | Y | Y | Can’t Tell | Can’t Tell | Y | Y | Y | **8** |
| Wagholikar Kb et col [7] | Y | Y | Y | Y | Y | Y | Y | Y | Y | Y | **10** |
| Wagholikar KB et col [8] | Y | Y | Y | Y | Y | Y | Y | Y | Y | Y | **10** |
| Sordo M et col [9] | Y | Y | Y | Can’t Tell | Can’t Tell | Can’t Tell | Can’t Tell | Y | Y | Y | **6** |
| Mehrabi S et col [10] | Y | Y | Y | Y | Y | Can’t Tell | Can’t Tell | Y | Y | Y | **8** |
| Kotfila C et col [11] | Y | Y | Y | Y | Y | Can’t Tell | Y | Y | Y | Y | **9** |
| Patterson OV et col [12] | Y | Y | Y | Y | Y | Can’t Tell | Can’t Tell | Y | Y | Y | **8** |
| Divita G et col [13] | Y | Y | Y | Y | Y | Can’t Tell | Can’t Tell | Y | Y | Y | **8** |
| Mei J et col [14] | Y | Y | Y | Y | Y | Can’t Tell | Can’t Tell | Y | Y | Y | **8** |
| Marco_Ruiz L et col [15] | Y | Y | Y | Y | Y | Y | Can’t Tell | Y | Y | Y | **9** |
| Danger R et col [16] | Y | Y | Y | Y | Y | Can’t Tell | Can’t Tell | Y | Y | Y | **8** |
| Breischneider et col [17] | Y | Y | Y | Y | Y | Can’t Tell | Can’t Tell | Y | Y | Y | **8** |
| Yang Z et col [18] | Y | Y | Y | Y | Y | Can’t Tell | Can’t Tell | Y | Y | Y | **8** |
| Wissel BD et col [19] | Y | Y | Y | Y | Y | Y | Y | Y | Y | Y | **10** |
| Wulff A et col [20] | Y | Y | Y | Y | Y | Can’t Tell | Can’t Tell | Y | Y | Y | **8** |
| Kulchak RA et col [21] | Y | Y | Y | Y | Y | Can’t Tell | Y | Y | Y | Y | **9** |
| Suh SH et col [22] | Y | Y | Y | Y | Y | Can’t Tell | Can’t Tell | Y | Y | Y | **8** |
| Park et col [23] | Y | Y | Y | Y | Y | Can’t Tell | Can’t Tell | Y | Y | Y | **8** |
| Afshar et col [24] | Y | Y | Y | Y | Y | Can’t Tell | Y | Y | Y | Y | **9** |

1. Was there a clear statement of the aims of the research?

2. Is a qualitative methodology appropriate?

3. Was the research design appropriate to address the aims of the research?

4. Was the recruitment strategy appropriate to the aims of the research?

5. Was the data collected in a way that addressed the research issue?

6. Has the relationship between researcher and participants been adequately considered?

7. Have ethical issues been taken into consideration?

8. Was the data analysis sufficiently rigorous?

9. Is there a clear statement of findings?

10. How valuable is the research?

Quality Appraisal – CASP Checklist for Systematic Review

|  | **1** | **2** | **3** | **4** | **5** | **6** | **7** | **8** | **9** | **10** | **Quality rating** |
| --- | --- | --- | --- | --- | --- | --- | --- | --- | --- | --- | --- |
| Ross MK et col [25] | Y | Y | Y | Y | Y | Y | Y | Y | Y | Y | 10 |
| van de Burgt BWM et col [26] | Y | Y | Y | Y | Y | Y | Y | Y | Y | Y | 10 |

1. Did the review address a clearly focused question?

2. Did the authors look for the right type of papers?

3. Do you think all the important, relevant studies were included?

4. Did the review’s authors do enough to assess quality of the included studies?

5. If the results of the review have been combined, was it reasonable to do so?

6. What are the overall results of the review?

7. How precise are the results?

8. Can the results be applied to the local population?

9. Were all important outcomes considered?

10. Are the benefits worth the harms and costs?

**References**

1. Clark C, Aberdeen J, Coarr M, Tresner-Kirsch D, Wellner B, Yeh A, et al. MITRE system for clinical assertion status classification. J Am Med Inform Assoc. 2011;18: 563. doi:10.1136/AMIAJNL-2011-000164

2. Patrick JD, Nguyen DHM, Wang Y, Li M. A knowledge discovery and reuse pipeline for information extraction in clinical notes. J Am Med Inform Assoc. 2011;18: 574. doi:10.1136/AMIAJNL-2011-000302

3. Roberts K, Harabagiu SM. A flexible framework for deriving assertions from electronic medical records. J Am Med Inform Assoc. 2011;18: 568. doi:10.1136/AMIAJNL-2011-000152

4. Jiang M, Chen Y, Liu M, Rosenbloom ST, Mani S, Denny JC, et al. A study of machine-learning-based approaches to extract clinical entities and their assertions from discharge summaries. J Am Med Inform Assoc. 2011;18: 601. doi:10.1136/AMIAJNL-2011-000163

5. D’Avolio LW, Nguyen TM, Goryachev S, Fiore LD. Automated concept-level information extraction to reduce the need for custom software and rules development. J Am Med Inform Assoc. 2011;18: 607. doi:10.1136/AMIAJNL-2011-000183

6. Garla V, Re V Lo, Dorey-Stein Z, Kidwai F, Scotch M, Womack J, et al. The Yale cTAKES extensions for document classification: architecture and application. J Am Med Inform Assoc. 2011;18: 614. doi:10.1136/AMIAJNL-2011-000093

7. Wagholikar KB, MacLaughlin KL, Henry MR, Greenes RA, Hankey RA, Liu H, et al. Clinical decision support with automated text processing for cervical cancer screening. J Am Med Inform Assoc. 2012;19: 833. doi:10.1136/AMIAJNL-2012-000820

8. Wagholikar Dr. KB, MacLaughlin KL, Kastner TM, Casey PM, Henry M, Greenes RA, et al. Research and applications: Formative evaluation of the accuracy of a clinical decision support system for cervical cancer screening. J Am Med Inform Assoc. 2013;20: 749. doi:10.1136/AMIAJNL-2013-001613

9. Sordo M, Rocha BH, Morales AA, Maviglia SM, Dell’Oglio E, Fairbanks A, et al. Modeling Decision Support Rule Interactions in a Clinical Setting. Stud Health Technol Inform. 2013;192: 908–912. doi:10.3233/978-1-61499-289-9-908

10. Mehrabi S, Schmidt CM, Waters JA, Beesley C, Krishnan A, Kesterson J, et al. An Efficient Pancreatic Cyst Identification Methodology Using Natural Language Processing. Stud Health Technol Inform. 2013;192: 822–826. doi:10.3233/978-1-61499-289-9-822

11. Kotfila C, Uzuner Ö. A systematic comparison of feature space effects on disease classifier performance for phenotype identification of five diseases. J Biomed Inform. 2015;58: S92. doi:10.1016/J.JBI.2015.07.016

12. Patterson O V., Forbush TB, Saini SD, Moser SE, Duvall SL. Classifying the Indication for Colonoscopy Procedures: A Comparison of NLP Approaches in a Diverse National Healthcare System. Stud Health Technol Inform. 2015;216: 614–618. doi:10.3233/978-1-61499-564-7-614

13. Divita G, Carter M, Redd A, Zeng Q, Gupta K, Trautner B, et al. Scaling-up NLP pipelines to process large corpora of clinical notes. Methods Inf Med. 2015;54: 548–552. doi:10.3414/ME14-02-0018/ID/JR0018-16

14. Mei J, Liu H, Li X, Xie G, Yu Y. A Decision Fusion Framework for Treatment Recommendation Systems. Stud Health Technol Inform. 2015;216: 300–304. doi:10.3233/978-1-61499-564-7-300

15. Marco-Ruiz L, Maldonado JA, Karlsen R, Bellika JG. Multidisciplinary Modelling of Symptoms and Signs with Archetypes and SNOMED-CT for Clinical Decision Support. Stud Health Technol Inform. 2015;210: 125–129. doi:10.3233/978-1-61499-512-8-125

16. Danger R, Corrigan D, Soler JK, Kazienko P, Kajdanowicz T, Majeed A, et al. A methodology for mining clinical data: experiences from TRANSFoRm project. Stud Health Technol Inform. 2015;210: 85–89. doi:10.3233/978-1-61499-512-8-85

17. Breischneider C, Zillner S, Hammon M, Gass P, Sonntag D. Automatic Extraction of Breast Cancer Information from Clinical Reports. Proc IEEE Symp Comput Based Med Syst. 2017;2017-June: 213–218. doi:10.1109/CBMS.2017.138

18. Yang Z, Huang Y, Jiang Y, Sun Y, Zhang Y-J, Luo P. Clinical Assistant Diagnosis for Electronic Medical Record Based on Convolutional Neural Network. ScIeNTIfIc REPORtS |. 2018;8: 6329. doi:10.1038/s41598-018-24389-w

19. Wissel BD, Greiner HM, Glauser TA, Holland-Bouley KD, Mangano FT, Santel D, et al. Prospective Validation of a Machine Learning Model that Uses Provider Notes to Identify Candidates for Resective Epilepsy Surgery. Epilepsia. 2020;61: 39. doi:10.1111/EPI.16398

20. Wulff A, Mast M, Hassler M, Montag S, Marschollek M, Jack T. Designing an openEHR-Based Pipeline for Extracting and Standardizing Unstructured Clinical Data Using Natural Language Processing. Methods Inf Med. 2020;59: e64. doi:10.1055/S-0040-1716403

21. Kulchak Rahm A, Walton NA, Feldman LK, Jenkins C, Jenkins T, Person TN, et al. User testing of a diagnostic decision support system with machine-assisted chart review to facilitate clinical genomic diagnosis. BMJ Health Care Inform. 2021;28. doi:10.1136/BMJHCI-2021-100331

22. Suh HS, Tully JL, Meineke MN, Waterman RS, Gabriel RA. Identification of Preanesthetic History Elements by a Natural Language Processing Engine. Anesth Analg. 2022;135: 1162–1171. doi:10.1213/ANE.0000000000006152

23. Park EH, Watson HI, Mehendale F V, O’Neil AQ, Clinical Evaluators. Evaluating the Impact on Clinical Task Efficiency of a Natural Language Processing Algorithm for Searching Medical Documents: Prospective Crossover Study. JMIR Med Inform. 2022;10: e39616. doi:10.2196/39616

24. Afshar M, Adelaine S, Resnik F, Mundt MP, Long J, Leaf M, et al. Deployment of Real-time Natural Language Processing and Deep Learning Clinical Decision Support in the Electronic Health Record: Pipeline Implementation for an Opioid Misuse Screener in Hospitalized Adults. JMIR Med Inform. 2023;11: e44977. doi:10.2196/44977

25. Ross MK, Wei W, Ohno-Machado L. “Big Data” and the Electronic Health Record. 2014. doi:10.15265/IY-2014-0003

26. van de Burgt BWM, Wasylewicz ATM, Dullemond B, Grouls RJE, Egberts TCG, Bouwman A, et al. Combining text mining with clinical decision support in clinical practice: a scoping review. Journal of the American Medical Informatics Association. 2023;30: 588–603. doi:10.1093/JAMIA/OCAC240
